# p130Cas Over-Expression Impairs Mammary Branching Morphogenesis in Response to Estrogen and EGF

**DOI:** 10.1371/journal.pone.0049817

**Published:** 2012-12-11

**Authors:** Maria del Pilar Camacho Leal, Alessandra Pincini, Giusy Tornillo, Elisa Fiorito, Brigitte Bisaro, Elisa Di Luca, Emilia Turco, Paola Defilippi, Sara Cabodi

**Affiliations:** 1 Molecular Biotechnology Center (MBC), Department of Genetics, Biology and Biochemistry, University of Turin, Turin, Italy; 2 Centre for Molecular Medicine Norway (NCMM), Nordic EMBL Partnership, Oslo, Norway; II Università di Napoli, Italy

## Abstract

p130Cas adaptor protein regulates basic processes such as cell cycle control, survival and migration. p130Cas over-expression has been related to mammary gland transformation, however the *in vivo* consequences of p130Cas over-expression during mammary gland morphogenesis are not known. In ex vivo mammary explants from MMTV-p130Cas transgenic mice, we show that p130Cas impairs the functional interplay between Epidermal Growth Factor Receptor (EGFR) and Estrogen Receptor (ER) during mammary gland development. Indeed, we demonstrate that p130Cas over-expression upon the concomitant stimulation with EGF and estrogen (E2) severely impairs mammary morphogenesis giving rise to enlarged multicellular spherical structures with altered architecture and absence of the central lumen. These filled acinar structures are characterized by increased cell survival and proliferation and by a strong activation of Erk1/2 MAPKs and Akt. Interestingly, antagonizing the ER activity is sufficient to re-establish branching morphogenesis and normal Erk1/2 MAPK activity. Overall, these results indicate that high levels of p130Cas expression profoundly affect mammary morphogenesis by altering epithelial architecture, survival and unbalancing Erk1/2 MAPKs activation in response to growth factors and hormones. These results suggest that alteration of morphogenetic pathways due to p130Cas over-expression might prime mammary epithelium to tumorigenesis.

## Introduction

p130Cas, originally identified as a highly phosphorylated protein in cells transformed by v-Src and v-Crk oncogenes, is a multifunctional adaptor protein required for embryonic development [1] and is characterized by structural motifs that enable interactions with a variety of signaling molecules. These multi-protein complexes sense and integrate signaling originating from several receptor systems [2]. In normal cells, p130Cas modulates cell motility, survival and proliferation [3]. p130Cas is emerging as an important player in the transformation and invasion driven by different oncogenes [4], [5]. In particular, we have previously shown that p130Cas accelerates mammary tumor formation and progression in the presence of ErbB2 [6], [7]. Moreover, it has been shown that patients with primary breast tumors expressing high levels of p130Cas (also known as BCAR-1) experience a more rapid disease recurrence and have a greater risk of resistance to tamoxifen therapy [8]. In addition, we have shown that in estrogen-dependent T47D breast cancer cells, p130Cas associates to the estrogen receptor alpha to form a macromolecular complex containing c-Src kinase, Crk, and p85 subunit of phosphatidylinositol 3-kinase (PI3K) and mediates non genomic estrogen signaling [9]. However, few data are addressing the i*n vivo* role of p130Cas in the mammary gland development. We have demonstrated in a MMTV-model of transgenic mice that the over-expression of p130Cas affects mammary gland development by inducing epithelial cell hyperplasia during pregnancy and lactation and delayed involution [10]. Nevertheless, neither functional nor mechanistic data on the role of p130Cas during hormonal and growth factors driven mammary gland morphogenesis have been reported.

At birth, the mammary gland consists of a simple ductal network that fills a fraction of the mammary fat pad and remains quiescent until puberty when steroid and pituitary hormones, local growth factors and cytokines, stimulate branching morphogenesis by inducing rapid proliferation and expansion of this primitive mammary network [11], [12]. The mammary tree expansion is driven by the terminal end-buds (TEBs) which are clover-shaped structures that encapsulate the tips of primary ducts. As primary ducts elongate, bifurcation (or primary branching) of the TEBs generates additional primary ducts, which in turn are subjected to lateral secondary branching, leading to tertiary lateral branches that occur at each diestrus and during pregnancy. When the extremities of the fat pad are reached, the end-buds shrink in size and become mitotically inactive, completing the pubertal growth phase [13]. The very complex process of branching morphogenesis is regulated by a wide range of factors expressed in the epithelium or stroma, such as epidermal growth factor (EGF), amphiregulin, hepatocyte growth factor (HGF) and fibroblast growth factor (FGF) [14], [15]. In addition, hormones including estrogen, progesterone, glucocorticoids, and retinoids have also been implicated in the development and maintenance of mammary epithelial structures [14], [16]. It has been shown that growth factors and hormones work in concert with each other to promote ductal morphogenesis *in vivo* [17]. Consistently, growth factor receptors such as Epidermal Growth Factor Receptor (EGFR) and Fibroblast Growth Factor Receptor (FGFR) families and estrogen receptor (ER) have been described to be crucial for normal mammary development [18], [19], [20], [21]

To examine the interplay between p130Cas, growth factor and estrogen signals, and mammary branching morphogenesis we choose, as experimental model, an organotypic 3D mammary primary culture that mimics aspects of mammary branching morphogenesis *in vivo* [22]. We demonstrated that p130Cas over-expression in presence of the concomitant stimulation with estrogen and EGF leads to aberrant mammary morphogenesis with the appearance of multicellular spherical filled structures characterized by impaired cell architecture and increased survival. The aberrant branching is supported by hyper-activation of Erk1/2 MAPKs and Akt signaling that is mainly dependent on ER. Our data indicate that during mammary morphogenesis, high levels of p130Cas expression can enforce EGFR/ER-induced proliferation and survival pathways that are hallmarks for cell transformation.

## Materials and Methods

### Mice

The use of animals was in compliance with the Guide for the Care and Use of Laboratory Animals published by the US National Institutes of Health. The protocol was approved by the Committee on the Ethics of Animal Experiments of the University of Torino on May 18^th^, 2011. All efforts were made to minimize animal suffering.

MMTV-p130Cas transgenic mice have been previously described [10]. Two independent MMTV-p130Cas transgenic lines were used (line 25 and line 37).

### Antibodies

Polyclonal antibodies to phospho-Erk1/2 MAPK (Thr202/Tyr204), phosphor-Src (Tyr416), phospho-p130Cas (Tyr410), p130Cas, Erk1/2 MAPK, phosphoAkt (S473) and Akt were purchased from Cell Signaling (Danvers, MA, USA). Antibodies against cleaved caspase-3 were from Millipore (Billerica, MA, USA). Antibodies against c-Src, Erk1/2, vinculin and tubulin from Santa Cruz (Palo Alto, CA, USA). Mouse monoclonal antibodies anti-Ki67 were from Novocastra, (Leica Microsystem, Germany), rabbit polyclonal antibodies anti-K14 from Covance (Pinceton, NJ, USA) and mouse monoclonal anti-keratin18 (K18) from (Progen, Heidelberg, Germany).

### Isolation of primary mammary organoids

4^th^ inguinal mammary glands were removed from 12 week-old virgin FVB-MMTV-p130Cas and wt littermates and minced. Minced tissue was incubated at 37°C for 40 minutes in a collagenase/trypsin mixture (0.25% trypsin-EDTA) (Gibco, Grand Island NY, USA), 0.2% collagenase type IV (Roche Applied Science, IN) in phenol red-free DMEM-F12 (Gibco) with 5% charcoal fetal bovine serum (Gibco Grand Island NY, USA), 5 ng/ml insulin, 50 ng/ml gentamicin, and 1% penicillin/streptomycin (Gibco) (basal medium). In order to isolate the epithelial content from the fat, the suspension obtained after digestion was centrifuged three times at 1500 rpm for 10 minutes and the supernatant was recovered. The final pellet was resuspended in Matrigel Phenol Red Free Growth Factor Reduced (BD Transduction Laboratories, Franklin Lakes, NY).

### Morphogenesis assays and Analysis of branching morphogenesis

Primary organoids were mixed in Matrigel and added to a 96-well containing an underlay of 25 µl of solidified Matrigel. Basal medium (phenol red-free DMEM-F12) supplemented with 35 ng/ml of Epidermal Growth Factor (EGF) or basic Fibroblast Growth Factor (FGF) (both from Sigma (St Louis, MI, USA)) and with or without 10 nM 17β-estradiol (E2) (Tocris, Park Ellisville, MO, USA) were added to the wells. Every other day, medium of all samples was replenished. The branching phenotype of organoids was determined after cultivation for 4–5 days. A branching phenotype was defined as an organoid having at least two branches extending from its central body, whereas secondary branching was defined as branches coming out from primary branches. Brightfield microscope images were acquired using the Axio Observer Z1 microscope (Zeiss, Germany). Pharmacological inhibitors for EGFR (1 µM AG1478, Sigma, St Louis, MI, USA), Estrogen Receptor (100 nM ICI 182,780, Tocris) and Erk1/2 MAPK (2 µM PD98059, Sigma, St Louis, MI, USA) were added to basal medium with or without EGF and 17β-estradiol stimuli 24 hours after plating and medium was replaced every other day. Branching response was evaluated after 4–5 days of culture as described above.

Quantification of branching was carried out by counting the percentage of branching in each well (50–100 organoids/well) All experiments were carried out at least in triplicates in at least 4 independent experiments.

### Organoids stimulation, protein extraction, SDS-Page and western blotting analysis

Phosphorylation kinetics of Erk1/2 MAPKs, Akt and c-Src were evaluated by adding growth factor/17β-estradiol supplemented medium for different time points. For inhibition of EGFR, ER and MAPK signaling, organoid cultures were left without stimuli for 24 hours followed by the addition of 1 µM AG1478, 100 nM ICI 182,780 and 2 µM PD98059 to basal medium for 1 hour at 37°C. For protein extraction, media were removed and organoids were washed once with cold PBS plus phosphatase inhibitors (NaVO_4_, 1 mM and NaF 1 mM) (Sigma, St Louis, MI, USA), and treated with the Cell recovery solution (BD Transduction laboratories, Franklin Lakes, NY) for 25 minutes at 4°C. The suspension obtained was centrifuged at 500 g for 5 minutes at 4°C. Supernatant was discarded and pellet containing organoids was then treated with a lysis buffer (2% SDS, 5 mM EDTA, 1 M Tris HCl pH 7.5) supplemented with protease inhibitor cocktail (Roche Applied Science, IN) and phosphatase inhibitors: 10 mM NaF, 1 mM sodium vanadate and 10 mM PMSF (Sigma, St Louis, MI, USA). Cell lysates were separated on 8% SDS-polyacrylamid gels and western blotting was performed.

### Immunofluorescence analysis

Immunofluorescence analysis was performed as described in Fata et al. (2007). Briefly, for detection of keratin-14 (K14), keratin-18 (K18) and Ki67, organoids were treated with a 18% sucrose solution in PBS for 15 minutes at RT followed by a 15 minute treatment with 30% sucrose in PBS at RT. After sucrose treatments, organoids were gently pipetted up and down and smeared onto a glass slide. Smeared organoids were air dried for 2 hours at RT and then fixed in methanol∶acetone (1∶1 v/v) for 10 minutes at −20°C. After fixation slides were air-dried at RT for 2 hours followed by blocking in 10% goat serum in PBS for 1 hour at RT. Primary antibodies were incubated in blocking buffer overnight at 4°C, followed by 3 washes of 15 minutes each in 0.5% Triton-X in PBS. Alexa dyes-coniugated secondary antibodies (Molecular Probes, Invitrogen) were used at 1∶1000 dilutions. To visualize DNA, fixed cells were stained with 4′,6-diamidino-2-dhenylindole (DAPI; Sigma). Images were captured by a HCX PL APO CS 63×1.4 OIL Leica TCS-SP5 II confocal microscope and analyzed with LASAF software (Leica, Germany).

### TUNEL assay

For the analysis of apoptotic cells, fragmentation of DNA in populations of cells in organotipic cultures was detected by using the in situ cell death detection kit TMR protocol (Roche Applied Science, IN). Briefly, after fixation of organoids as described above, smeared organoids onto glass slide were washed twice with PBS and samples were permeabilized with Triton 0.5% for 2 minutes on ice. Organoids were washed 3 times with PBS, and incubated with the TUNEL-reaction mixture (Roche Applied Science, Indianapolis, IN, USA) for 60 minutes at 37°C. Additional PBS washes were done before the analysis of the samples by fluorescence microscopy. Images were acquired using an ApoTome system (Zeiss, Germany).

### Statistical analyses

Statistical significance was determined with two tailed Student's t-test with error bars representing standard error of the mean (SEM). Statistical significance was also evaluated by using Anova two-way test. p<0.05 (*) p<0.001 (**).

## Results

### p130Cas expression enhances EGF-mediated branching morphogenesis in mouse primary organoids

**Figure 1:**
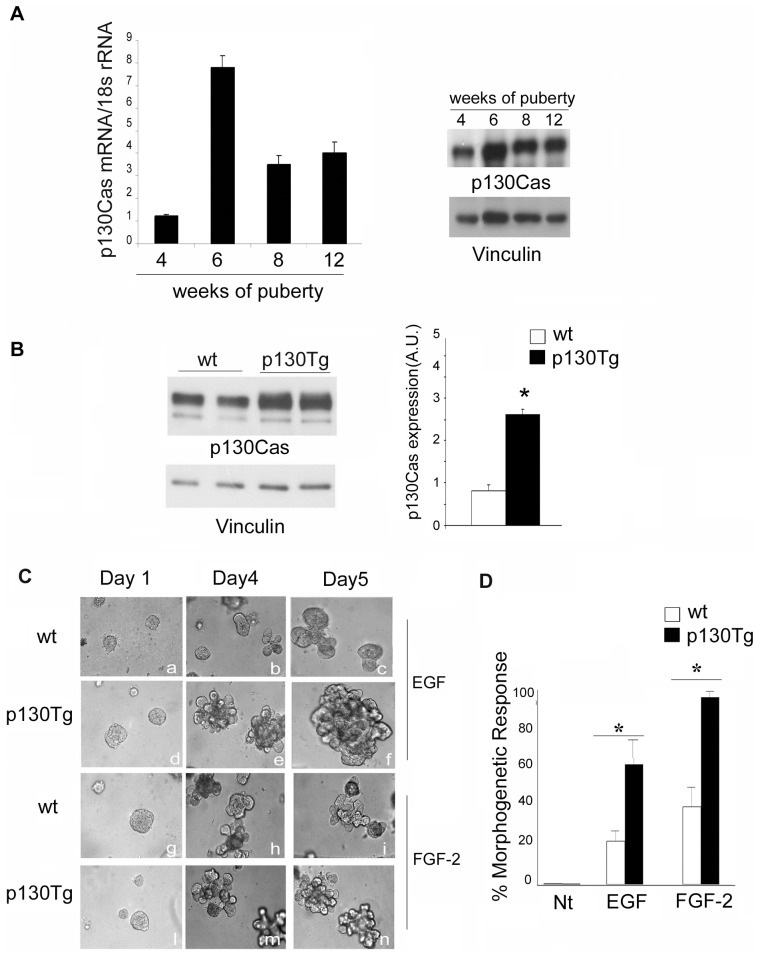
Effects of p130Cas expression on branching morphogenesis in primary mouse mammary gland organoids grown in 3D Matrigel cultures. (A) Let panel: qRT-PCR expression analysis of p130Cas using total RNA isolated from the 4th inguinal mammary gland of at least three FVB wt mice during different stages of puberty. The amount of p130Cas mRNA was normalized to 18S rRNA. Right panel: Analysis of p130Cas protein expression from wt mice (three mice/stage) at the same stages of puberty as in the left panel. (B) Analysis of p130Cas protein expression from 5 day untreated wt and p130Tg organoids (upper panel) and quantification analysis expressed as the mean of p130Cas/tubulin ± standard deviation (SD) (*p<0.05) (lower panel). (C) Brightfield images of representative wt and p130Tg organoids, after 1, 4 and 5 days of culture in presence of EGF (35 ng/ml) or FGF-2 (35 ng/ml) in the culture medium. Images were captured by using the Axio Observer Z1 microscope. (D) Quantitative analyses of branching morphogenesis after treatment of wt and p130Tg organoids with EGF and FGF-2 at 5 days. The plot represents the mean number of overall branching response in at least three wells from 4 independent experiments. Similar results were obtained with a second p130Cas transgenic line (data not shown). Quantification of the mean number of branched organoids buds was significantly higher in EGF and FGF-2 treated p130Tg organoids compared to wt organoids (*p<0.002).

We have previously shown in a MMTV transgenic model that p130Cas over-expression in the mammary gland leads to hyperplasia of the mammary epithelium during pregnancy and lactation and to delayed involution [10]. However, the role of p130Cas during the early phases of mammary gland development is still unknown. To address this point, we first evaluated the levels of expression of p130Cas in physiological conditions by performing real-time reverse transcriptase PCR (qPCR) on total RNAs isolated from wt primary mammary epithelial cells of FVB mice at different times during puberty. The results indicate that the levels of p130Cas mRNA in the wt mammary gland peaks during puberty at 6 weeks of age (Fig. 1A, right panel), suggesting a biological role for p130Cas in pubertal mammary development in virgin mice. Consistently, p130Cas protein also show a similar trend of expression at the same developmental stages (Fig. 1A, left panel). To explore the mechanism implicating p130Cas during branching morphogenesis, we prepared organotypic 3D mammary primary cultures from MMTV-p130Cas transgenic mice. This culture model mimics aspects of mammary branching morphogenesis *in vivo* and is responsive to growth factors and hormones produced in the mammary gland [22]. Mammary gland organoids were isolated from 12 week old wt and p130Cas-MMTV transgenic mice and allowed to grow in matrigel. At this age, high yield organoid preparations can be easily obtained [22] and p130Cas protein expression is elevated. p130Cas expression was evaluated in wt and transgenic organoids and, as expected, it was about 2.5 fold higher in p130Cas organoids (p130Tg) compared to the wt (Fig. 1B, upper and lower panels). Mammary branching was induced by stimulating the cell culture with FGF-2 and EGF, two growth factors implicated in the local control of postnatal mammary development [11], [15], [20], [21], [23], for the indicated days (Fig. 1C). Interestingly, the over-expression of p130Cas itself did not affect mammary branching (Fig. S1A). Conversely, the addition of EGF and FGF-2 led to an altered morphogenetic response in p130Tg organoids characterized by a strong increase in primary and secondary branching at 4 and 5 days (Fig. 1C, panels e, f, m, n) compared to wt organoids (Fig. 1C, panels c, d, h, i). Specifically, as a measure of the morphogenetic response, we counted the relative number of organoids with primary and secondary branching, regardless of their size, and we showed that p130Tg organoids at 5 days of culture show a 3 fold increase in the morphogenetic response upon EGF stimulation and a 2.3 fold increase upon FGF-2 stimulation compared to the wt (Fig. 1D). These results ex-vivo support the involvement of p130Cas during growth-factor dependent branching morphogenesis.

### p130Cas over-expression impairs branching morphogenesis of mammary organoids in response to estrogen and EGF stimulation

**Figure 2:**
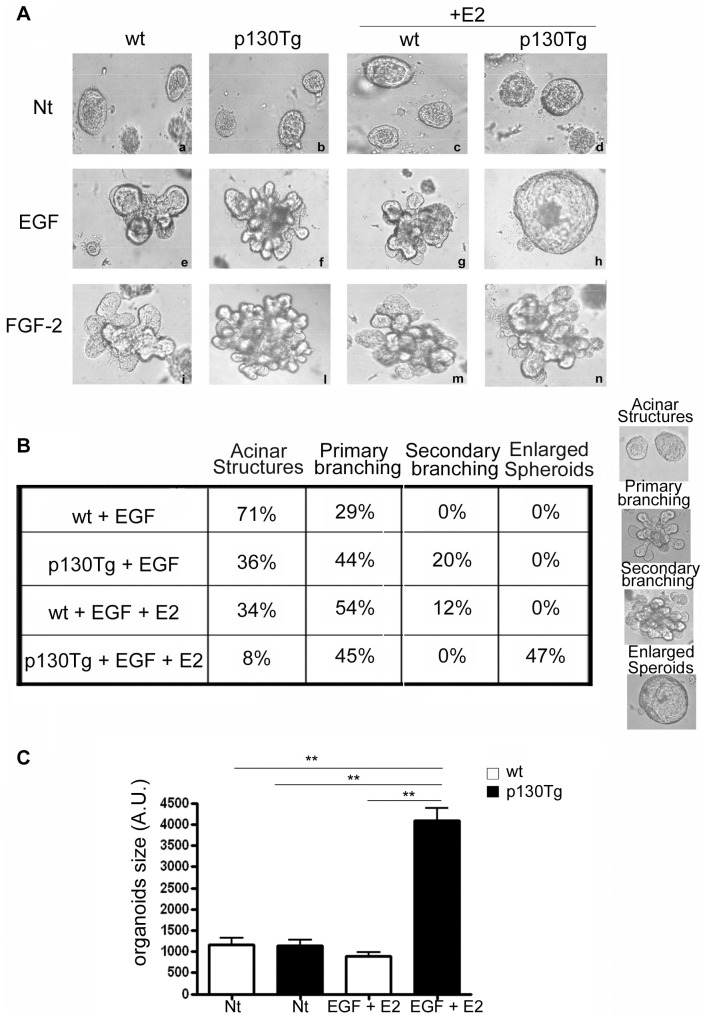
Effects of E2 stimulation on EGF− and FGF-dependent branching morphogenesis. (A) Brightfield images of representative wt and p130Tg organoids, cultured for 5 days in the absence or presence of E2, EGF, or the combination of both. (B) Quantification of primary and secondary branching of wt vs. p130Tg organoids upon treatment with either E2, EGF or EGF+E2 for 5 days. Experiments were repeated at least five times. Two-way ANOVA analysis show a significant effect of both treatment (EGF vs. EGF+E) and genotype (wild type vs. transgenic) for Acinar Structures, secondary branching and enlarged spheroids (P<0.0001 for both treatment and genotype). For primary branching only treatment has a significant effect (P<0.0001). Interaction between treatment and genotype is significant for all variables (Acinar: P<0.0065; primary branching: P<0.0001; secondary branching: P<0.0001; enlarged spheroids: P<0.0001). (C) Quantification of spherical organoids size in untreated and EGF+E2 treated wt and p130Tg organoids was performed by using ImageJ software. Statistical significance is indicated (** p<0.001).

In the pubertal mammary gland, the initial driving force for ductal morphogenesis comes from circulating ovarian and pituitary hormones. In particular, there are critical requirements for estrogen during ductal morphogenesis [14], [16], [24]. Moreover, p130Cas scaffold protein has been described as a highly dynamic component of the estrogen receptor signaling complex and plays a crucial role in the early steps of estrogen-dependent non-genomic signaling in breast cancer cells [9]. Therefore, we assessed whether high levels of p130Cas upon estrogen stimulation affect mammary gland development. Morphogenesis of wt and p130Cas organotypic cultures was evaluated in the presence of estrogen alone (E2) and in combination with growth factors. The addition of E2 in the primary cultures did not alter the morphogenetic response regardless of p130Cas over-expression (Fig. 2A, panels c–d). Surprisingly, upon the concomitant stimulation with E2 and EGF, the over-expression of p130Cas promoted a unique phenotype characterized by the presence of spherical epithelial structures (Fig. 2A, panel h) whereas in wt organoids branching morphogenesis normally occurred (panel h). This was not due to an altered p130Cas protein expression upon E2, EGF and EGF+E2 treatments (Figure S1B). The transgenic phenotype was specific for the concomitant stimulation with E2 and EGF in presence of p130Cas, since branching morphogenesis was not affected in E2+FGF-2 stimulated organotypic cultures in both wt and p130Tg organoids (Fig. 2A, panels m,n). The quantitative analysis of the branching response indicated that the concomitant addition of EGF and E2 promoted primary and secondary branching in wt but not in p130Tg organoids where the formation of enlarged spherical epithelial structures was evident in approximately 50% of the organoids (Fig. 2B). These enlarged spherical structures were about four fold bigger compared to not-branched acinar structures observed in untreated wt and p130Tg organoids and in not branched EGF+E2 treated wt organoids (Fig. 2C). These data show that high levels of p130Cas in presence of EGF and E2 stimulation severely alters mammary gland morphogenesis leading to a specific phenotype characterized by absence of branching. Moreover, this altered morphogenesis is driven by combined E2 and EGF stimulation, suggesting that p130Cas preferentially interferes with responses generated by EGFR and ER rather than FGFR and ER.

### p130Cas-overexpression leads to impairment of epithelial architecture and luminal clearance in response to E2 and EGF treatment

**Figure 3:**
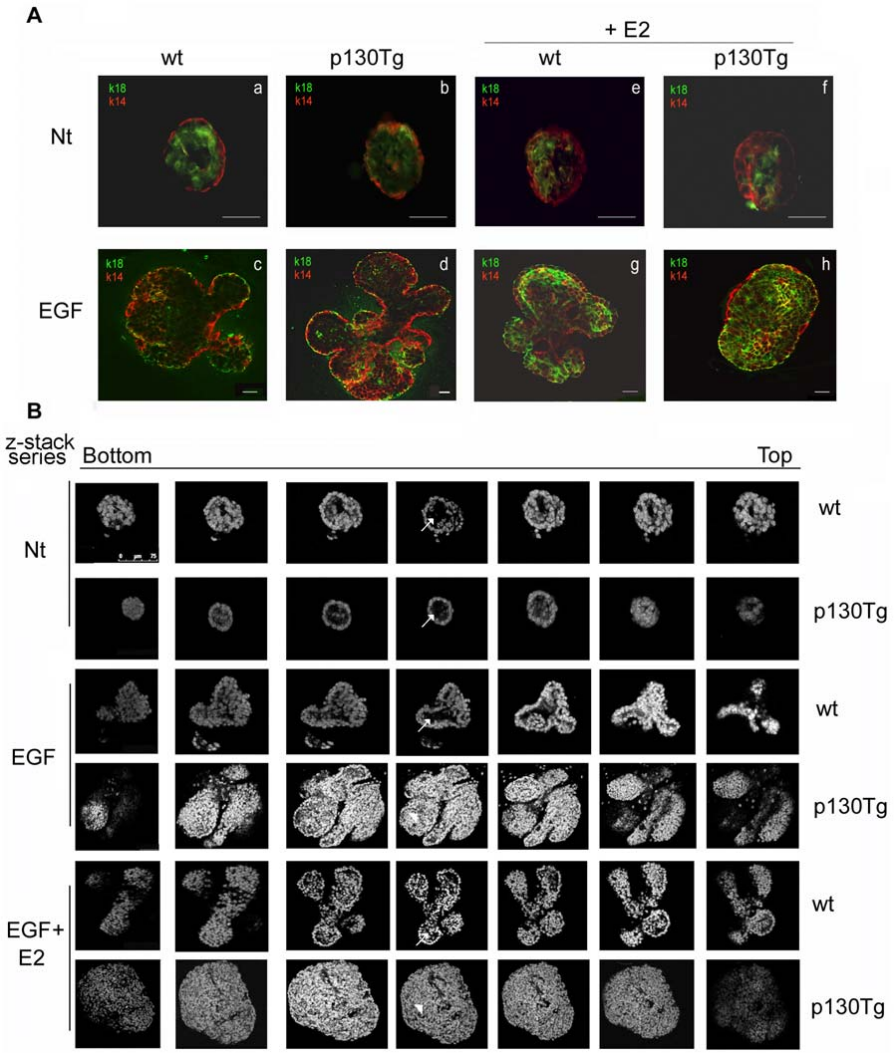
E2 treatment of p130Tg organoids alters myoepithelial-luminal architecture and lumen clearance. (A) Confocal images representing immunofluorescence staining for K14 (red; myoepithelium) and K18 (green; luminal epithelium) of wt and p130Tg organoids stimulated with EGF and E2 at day 5 of culture. Scale bar = 0,50 micron. (B) Confocal z-stacks of DAPI staining of wt and p130Tg organoids at day 5 of culture to visualize lumen clearance. Images in A and B were acquired using a Leica TCS-SP5 II confocal microscope. Images in A and B are representative of four independent experiments.

To determine whether the aberrant branching phenotype observed in the organoids isolated from the p130Tg mice was due to alterations in polarization and luminal cavitation, we performed immunofluorescence analysis by using antibodies against K14 and K18, which detect myoepithelial and luminal epithelial cells, respectively. Fluorescence microscopy analysis of K14 and K18 immunostaining after 4 days of culture (Fig. 3A) showed that untreated wt and p130Tg organoids preserved a basal layer of K14-positive myoepithelial cells and an inner layer of K18-positive luminal epithelial cells, a localization that resembles the *in vivo* mammary epithelial architecture (Fig. 3A, a–b). Wt and p130Tg organoids that have undergone EGF-dependent branching morphogenesis had similar spatial arrangement characterized by myoepithelial cells surrounding an inner luminal layer with developing branches and central lumen (Fig. 3A, c–d and Fig. S2). Strikingly, upon the concomitant addition of E2 and EGF, p130Tg organoids display a disorganized localization of K14-positive myoepithelial cells that were often present within the inner K18-positive cell layer (Fig. 3A, panel h and Figure S2) in contrast to wt-organoids that retained the bilayer organization of myoepithelial-luminal cells (Fig. 3A, panel g and Figure S2). To determine whether this altered architecture was due to defects in lumen cavitation, cells from EGF−, EGF+E2-treated and from untreated wt and p130Tg organoids were immunostained with the nuclear marker DAPI and confocal microscopy images of z-stack were analyzed (Fig. 3B). The presence of the lumen was clearly visible in wt and p130Tg untreated organoids (Fig. 3B, first two rows, white arrows) and in EGF or EGF+E2-treated wt organoids (Fig. 3B, 3^rd^ and 5^th^ rows, white arrows). In contrast in p130Tg mammary organoids the lumen was partially filled upon EGF (Fig. 3B, 4^th^ row, white arrowhead) or completely filled upon EGF+E2 (Fig. 3B, 6^th^ row, white arrowhead) stimulation. Thus, these data demonstrate that during EGF-branching morphogenesis p130Cas over-expression affects myoepithelial and luminal epithelial cell localization and that the concomitant stimulation with E2 leads to impairment of branching morphogenesis characterized by inhibition of lumen cavitation.

### p130Cas alters cell survival in EGF+E2-treated mammary organoids

**Figure 4:**
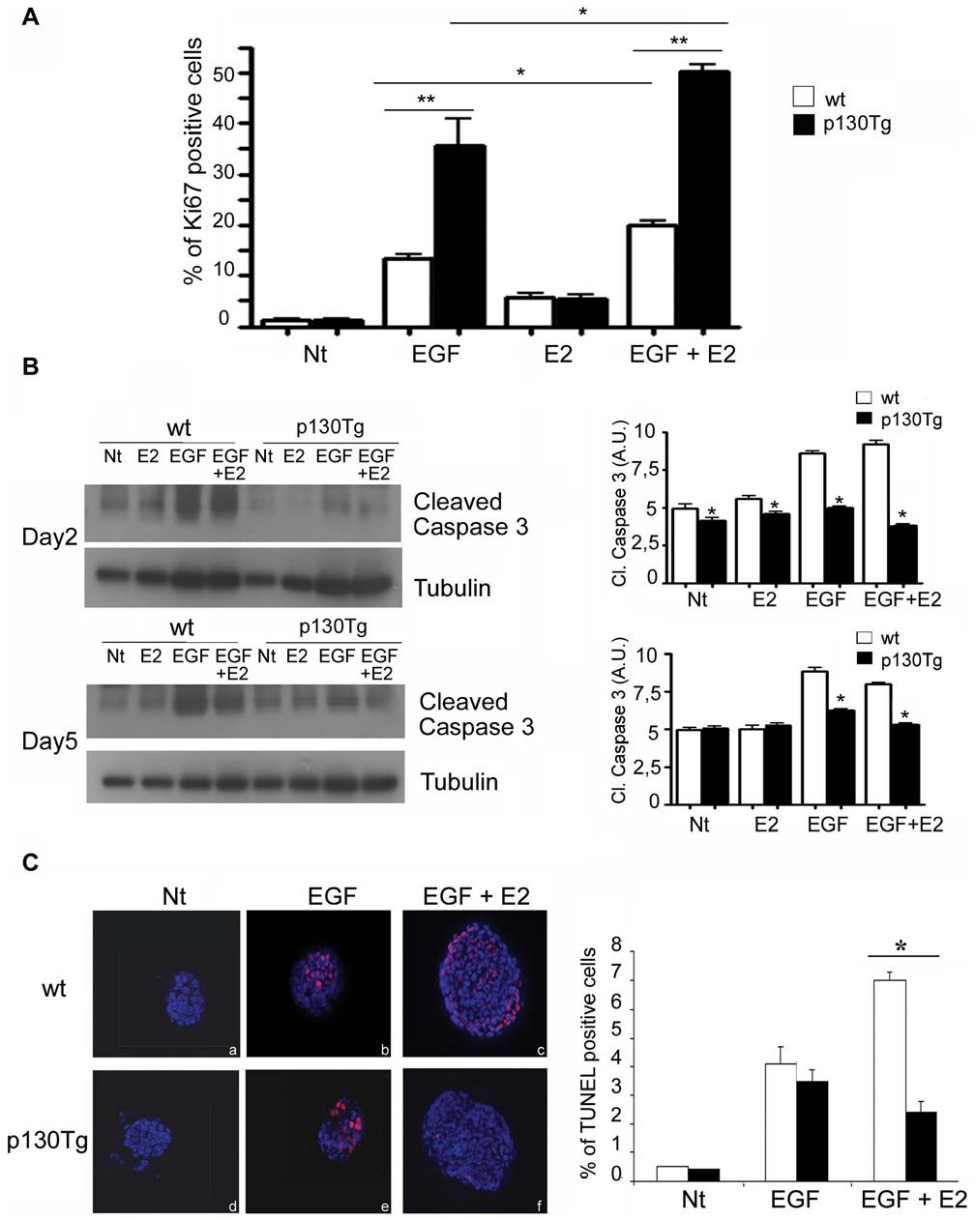
p130Cas over-expression enhances cell proliferation and survival in EGF and EGF+E2-treated primary organoids. (A) Quantification of nuclear Ki67 staining from total nuclei count of each organoid at day 2. Quantification analysis of Ki67 staining as mean of Ki67 positive cells/total number of cells ±SD and significance are reported (*p<0.05, **p<0.001). More than 20 organoids from each condition were counted in three independent experiments. (B) Western blot analysis of cleaved caspase-3 from 2 and 5 day of EGF, E2 and EGF+E2-treated wt and p130Tg cultured organoids. Quantification analysis at day 2 and day 5 of cleaved-caspase 3 are reported on the right (*p<0.05). (C) TUNEL analysis from 2 day treated wt and p130Cas cultured organoids. TUNEL positive cells in the organoids are stained in red. The plot represents the quantification of the results of three independent experiments in which apoptotic cells are counted and normalized to the total number of nuclei/organoid for at least 10 organoids from each condition ± standard deviation (*p<0.05).

An essential role for apoptosis during lumen formation has been previously established by using a non-tumorigenic human mammary epithelial cell line MCF10A which forms organized structures with hollow central lumens when grown in a 3D matrix [25]. In vivo, the mammary gland primary ducts behind the TEBs are characterized by a single layer of luminal epithelial cells surrounding an empty hollow lumen. The body cells that border the TEB and the luminal space possess high rates of apoptosis, which contrasts with the low apoptotic rates observed in cap cells at the distal end of the developing duct, suggesting that apoptosis of body cells may contribute to lumen formation [26]. Moreover, proliferation-dependent mechanisms that control lumen size have been described [26] and the role of p130Cas in cell survival and proliferation has been established in various in vitro and in vivo models [5]. Therefore, we assessed whether the impairment of mammary branching and lumen formation observed in the EGF+E2-treated p130Tg organoids was dependent on an aberrant proliferation and/or apoptosis rate. We evaluated proliferation levels in wt and p130Tg organoids by performing Ki67 immunostaining at 2 days of culture. While, there were no differences in cell proliferation between E2 treated wt and p130Tg organoids (Fig. 4A), cell proliferation was induced in EGF− and EGF+E2-treated wt and further induced in p130Tg organoids. The quantitative analyses of these data indicate that the extent of cell proliferation was slightly increased (p<0.02) in EGF+E2-treated p130Tg organoids compared to EGF+E2-treated wt organoids, thus indicating that the increased proliferation might contribute at least in part to altered branching. Therefore, we evaluated whether the alteration in branching morphogenesis observed was also due to differences in cell survival. As shown in Figure 4B (upper panel), at day 2 of culture, p130Cas over-expressing organoids in presence of EGF+E2 showed a marked decrease in the levels of cleaved caspase-3 compared to wt organoids. Significant differences in the cleavage of caspase-3 were also observed at day 5 of culture (lower panel). To further support this observation, we performed TUNEL analyses at day 2 of culture (Fig. 4C, left panel and right panel). TUNEL-positive nuclei were increased in both EGF treated wt and p130Tg organoids (Fig. 4C, panels b and e), whereas addition of E2 did not affect apoptosis (data not shown). Consistently with cleaved caspase 3 levels, in presence of concomitant stimulation with EGF+E2, the number of TUNEL positive nuclei was strongly reduced in p130Tg compared to wt organoids (Fig. 4 panels c and f). Overall, these data indicate that p130Cas over-expression at early times of culture protects from apoptosis in presence of E2 and EGF stimulation.

### p130Cas over-expression leads to Erk1/2 MAPK and Akt hyperactivation in response to EGF+E2 treatment of primary mammary organoids

**Figure 5:**
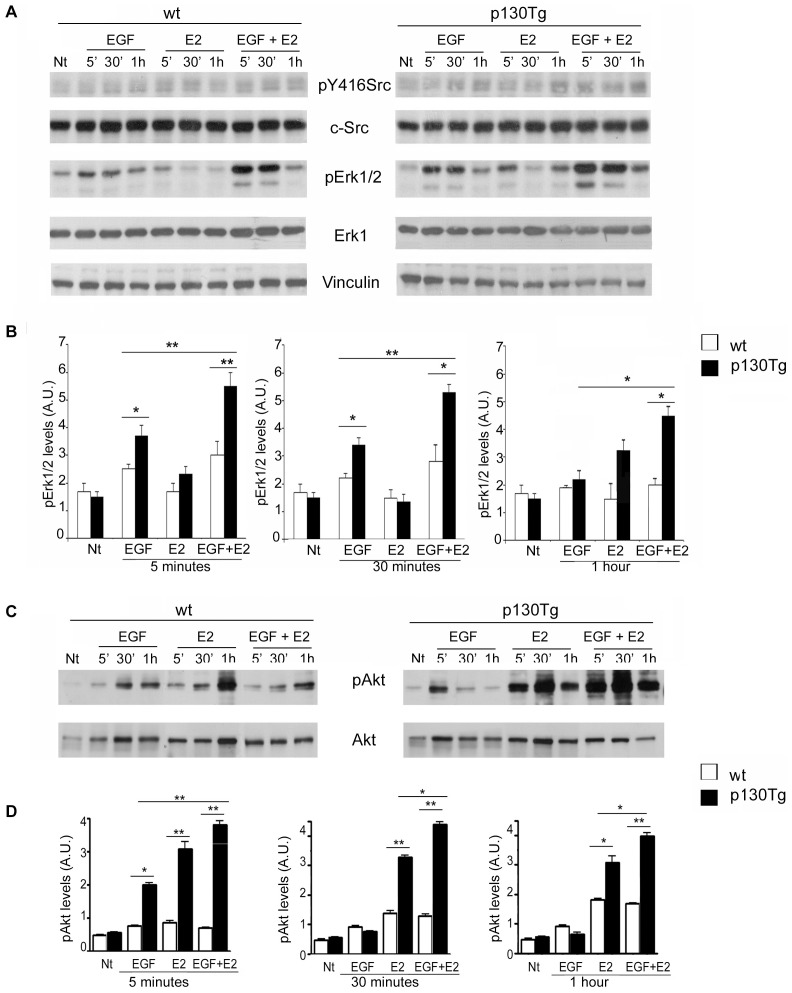
EGF+E2 treatment leads to Erk1/2 MAPKs and Akt hyperactivation in p130Tg organoids. (A) Western blot analysis of phospho-Erk1/2 MAPKs in wt or p130Tg organoids stimulated with EGF and EGF+E2 at the indicated times. Vinculin and total Erk1/2 MAPKs blots are provided as loading controls. Blots are representative of five independent experiments. (B) Quantification analysis of Erk1/2 MAPKs activation following stimulation for 5, 30 minutes and 1 hour. Data are represented as the mean of phospho-Erk1/2 MAPK levels/total Erk1/2 MAPK ± standard deviation (SD) of five independent experiments (*p<0.05). (C) Western blot analysis of phospho-Akt in wt or p130Tg organoids stimulated as in (A). Total Akt blots are provided as loading controls. Blots are representative of three independent experiments. (D) Quantification analysis of Akt activation following stimulation as in (A). Data are represented as the mean of phospho-Akt levels/total Akt ± standard deviation (SD) of three independent experiments (*p<0.05).

Since extracellular signal regulated kinase 1/2, mitogen-activated protein kinases (Erk1/2 MAPK) have been described to be master regulator of mammary gland morphogenesis by controlling both cell proliferation and apoptosis [22], we asked whether p130Cas over-expression was affecting their activation in response to E2 and EGF in the mammary organotypic model. After 2 days of culture both wt and p130Tg organoids were stimulated at the indicated times with EGF, E2, and EGF+E2 (Fig. 5A). Biochemical analysis of protein extracts of wt and p130Tg organoids showed that in p130Cas over-expressing organoids, EGF enhances Erk1/2 MAPKs phosphorylation levels compared to wt (Fig. 5A). Interestingly, the concomitant treatment with EGF and E2 led to a further Erk1/2 MAPKs activation in p130Cas-Tg with respect to wt organoids (Fig. 5A). Quantification of Erk1/2 MAPKs activation at 5, 30 minutes and 1 hour of stimulation is shown in Fig. 5B. Overall, these data show that high levels of p130Cas in mammary organoids result in increased levels and duration of Erk1/2 MAPKs activation. Our data show that Erk1/2 MAPKs activation observed in EGF+E2 is not dependent by differential activation of c-Src (Fig. 5A) and p125Fak or on EGFR stability between p130Tg and wt organoids (data not shown). These data indicate that the EGFR/Src/Fak axis does not correlate with Erk1/2 MAPKs hyperactivation.

To further characterize the signaling pathways that might contribute to both proliferation and apoptosis, we evaluated the activation of Akt, as the PI3K/Akt pathway has been described to be stimulated by both EGF and estradiol in breast cancer cells [27], [28]. Wt and p130Tg were cultivated for 2 days and stimulated as described above. While in EGF-treated p130Tg organoids Akt activation peaks at 5 minutes and then decreases, in E2 and EGF+E2-treated p130Tg organoids Akt activation is strongly and significantly enhanced and sustained compared to wt organoids (Fig. 5C and D). These data indicate that p130Cas over-expression leads to a synergistic effect of EGF and E2 on Akt activation. Taken together, these results indicate that both proliferative and survival pathways are strongly activated in EGF+E2-treated p130Tg compared to wt organoids.

### ER-dependent Erk1/2 MAPK activation levels are determinant for p130Cas-mediated branching morphogenesis

**Figure 6:**
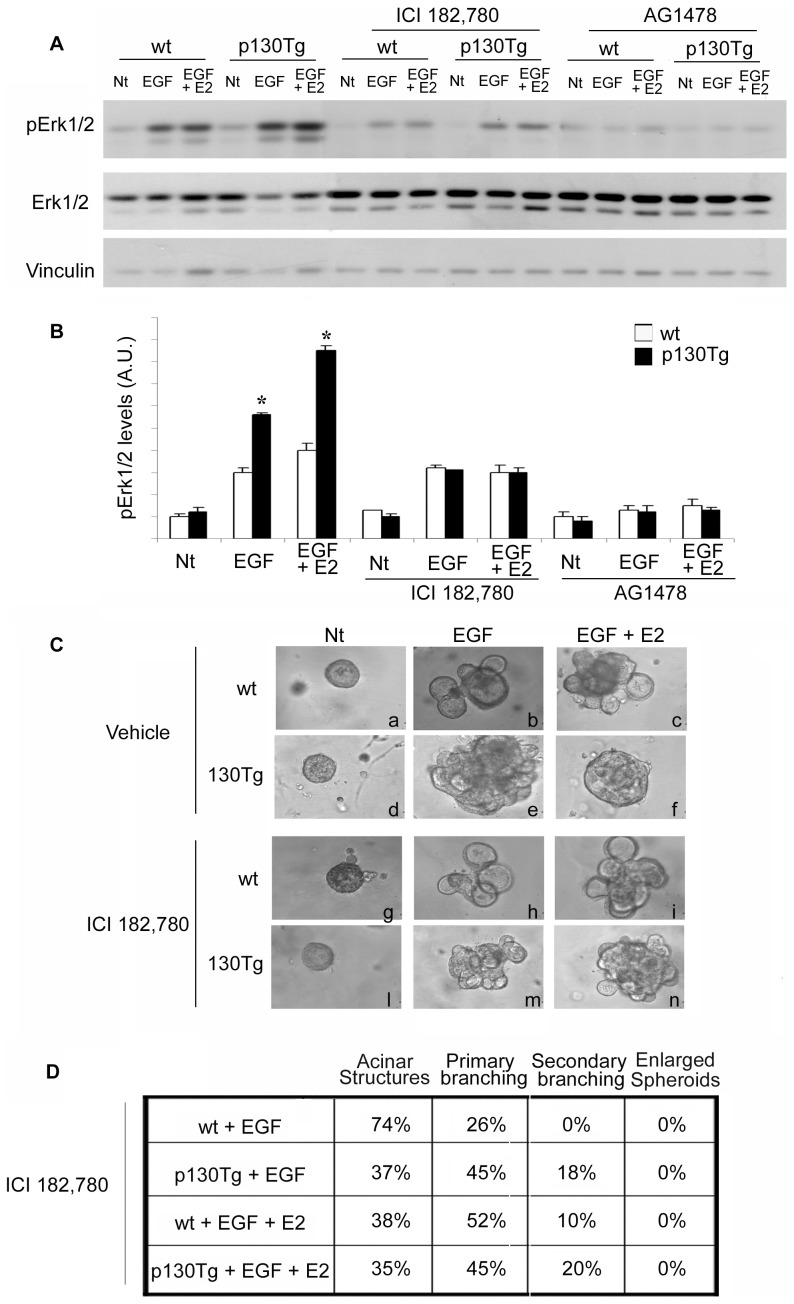
Inhibition of ER activity in EGF+E2-treated p130Tg mammary organoids prevents Erk1/2 MAPK hyper-activation and rescues branching morphogenesis. (A) Western blot analysis of phospho-Erk1/2 MAPKs in wt or p130Tg organoids pre-treated for 1 hour with ICI 182,780 or AG1478 followed by 5 minutes of EGF or EGF+E2 stimulation. Vinculin and total Erk1/2 MAPKs blots are provided as loading controls. Blots are representative of three independent experiments. (B) Quantification analysis of Erk1/2 MAPKs activation of wt and p130Tg organoids pre-treated for 1 hour with ICI 182,780 or AG1478 followed by 5 minutes of EGF or EGF+E2 stimulation is shown. Data are represented as the mean of the ratio of phospho-Erk1/2 MAPK and total Erk1/2 MAPK levels ± standard deviation (SD) (*p<0.05) of three independent experiments. (C) Brightfield images of representative wt and p130Tg organoids at 5 days of culture treated with ICI 182,780 and stimulated with EGF or EGF+E2 every other day. Images are representative of three independent experiments. (D) Quantification of primary and secondary branching of wt vs. p130Tg organoids at 5 days of culture treated with ICI 182,780 and stimulated with EGF or EGF+E2 of three independent analyses. Two-way ANOVA analysis show a significant effect of both treatment (EGF vs. EGF+E) and genotype (wild type vs. transgenic) for Acinar Structures, primary and secondary branching (P<0.0001 for both treatment and genotype). Interaction between treatment and genotype is significant for all variables (all P<0.0001).

To understand which signaling pathways is specifically involved in the p130Cas-dependent alteration of Erk1/2 MAPKs activation, wt and p130Tg organoids were treated with ER antagonist (ICI 182,780) or EGFR inhibitor (AG1478). Specifically, wt and p130Tg organoids at 2 days of culture were pre-treated with ICI 182,780 and AG1478 for 1 hour prior to 5 minutes of EGF or EGF+E2 stimulation. Protein extracts were analyzed for Erk1/2 MAPKs phosphorylation levels (Fig. 6A) and quantitative analysis was performed (Fig. 6A and 6B). The inhibition of EGFR activity in both wt and p130Tg organoids decreased Erk1/2 MAPKs activation to levels comparable to untreated organoids (Fig. 6A and 6B). Interestingly, interfering with ER signaling in wt organoids did not affect substantially Erk1/2 MAPKs activation upon EGF and EGF+E2 treatment. In contrast, ER inhibition in p130Tg organoids reduced in both conditions Erk1/2 MAPKs activity to levels similar to those observed in wt organoids (Fig. 6A, B). These data indicate that Erk1/2 MAPKs increased activity observed in p130Cas over-expressing organoids depends on ER stimulation. Since it has previously demonstrated that antagonizing ER activity by prolonged tamoxifen treatment stimulates adhesion dependent p130Cas tyrosine phosphorylation in breast cancer cell lines [29], we checked whether treatment with ICI 182, 780 and AG1470 affects p130Cas phosphorylation status. As shown in Figure S3, p130Cas phosphorylation levels were not affected by ICI 182,780. To address whether the re-establishment of the Erk1/2 MAPK activity to normal level following ER inhibition is sufficient to rescue branching morphogenesis in p130Tg organoids, we inhibited ER, EGFR, and Erk1/2 MAPKs in organoids and we evaluated the morphological outcome after 5 days of culture in the presence of EGF+E2 (Fig. 6C). The results showed that in both wt and p130Tg EGF− and EGF+E2-treated organoids, branching morphogenesis was completely blocked by MAPK and EGFR inhibition by using PD98059 and AG1478 (Fig. S4). ER inhibition by ICI 182,780 treatment led only to a slight reduction in branching in EGF or EGF+E2 treated wt organoids (Fig. 6C, compare panel b to h and c to i). Interestingly, in p130Tg organoids, the inhibition of ER in the presence of EGF+E2 abrogated the formation of enlarged multicellular epithelial spheroids re-establishing mammary branching (Fig. 6C, compare panel n to f). The quantitative analysis of the branching response indicated that ER inhibition by ICI 182,780 in presence of EGF+E2 stimulation rescued primary and secondary branching in p130Tg organoids to an extent that is similar to the EGF−stimulated 130Tg organoids (Fig. 6D). Overall, these data clearly show that p130Cas-dependent ER signaling impinges on Erk1/2 MAPKs activation levels impairing proper branching morphogenesis. Notably, re-establishing proper Erk1/2 MAPKs activity is sufficient to revert p130Cas-dependent aberrant branching morphogenesis.

## Discussion

Here, by taking advantage of mammary organoids, a physiologically relevant 3D model for studying mammary morphogenesis [22], we demonstrate the involvement of p130Cas in this process. The initial observation that during mammary development p130Cas expression peaks at 6 weeks of age and remains elevated during puberty, prompted us to investigate the role of p130Cas over-expression in mammary morphogenesis. Our results show that high levels of p130Cas expression during puberty enhances EGF-dependent mammary branching providing the first evidence of the involvement of this adaptor protein in this physiological process. As mentioned above, mammary morphogenesis is strictly dependent during puberty on growth factors and hormones stimulation [15]. Interestingly, upon the concomitant stimulation of EGF and E2, p130Cas over-expression dramatically affects mammary branching leading to the formation of enlarged spheroids with filled lumen, absence of branching, increased survival and hyper-activation of Erk1/2 MAPKs and Akt. In these conditions, interfering with ER activity is sufficient to re-establish normal branching, Erk1/2 MAPK indicating that p130Cas is a crucial regulator of ER and growth factor signaling during mammary morphogenesis.

The aberrant spheroids structures observed in p130Cas over-expressing organoids show an altered architecture, an increased survival as demonstrated by TUNEL analysis and cleaved caspase 3 expression. Several studies have pointed out the crucial role of apoptosis during the lumen formation [30], [31]. Moreover, it has been demonstrated that the lack of survival signals from ECM can trigger anoikis [32], [33] both *in vivo* in animal models and in 3D culture *in vitro* and that aberrant activation of growth factor receptors and hormone receptors can disrupt ductal architecture resulting in filling of the luminal space [25]. However, very little is known about the molecular mechanisms that disrupt normal ductal organization of epithelial cells. On the basis of our data, we can speculate that the decreased apoptosis observed in p130Cas over-expressing organoids can be due to the ability of p130Cas to convey multiple survival signaling originating from Extracellular Matrix, EGFR and ER [5]. Specifically, in branching morphogenesis, high levels of p130Cas coordinates and amplify survival signals originated from both ER and EGFR that ultimately lead to impairment of lumen formation.

Our data also indicate that in presence of high levels of p130Cas upon EGF and E2 stimulation, a hyper-activation of Erk1/2 MAPKs and Akt occurs. It has been previously reported that sustained levels of Erk1/2 activation upon TGF alpha rather than FGF7 stimulation, play a crucial role during pubertal mammary gland development and branching, suggesting that the mammary epithelium responds to multiple and concomitant growth factor signals by finely temper the extent and the duration of the MAPKs signals [22]. By antagonizing ER and by inhibition of EGFR activity, we were able to demonstrate that p130Cas affects the morphogenetic outcome by modulating the MAPK activation levels. Specifically, the treatment with the ER antagonist ICI 182,780 was able to rescue mammary branching in p130Tg organoids. Moreover, our data indicate that in presence of p130Cas, upon the concomitant treatment with EGF and E2, Akt activation is enhanced with respect to EGF alone and to wt organoids, thus underlining the role of p130Cas in promoting an additive effect of EGF and E2 stimulation on Akt activation. Therefore, these results suggest that p130Cas might be able to converge signals coming from different stimuli on downstream effector molecules thus leading to their abnormal activation.

It is now well accepted that E2 induces cellular effects independently on ER transcriptional activity both in vivo and in vitro [34], [35], [36]. These so-called non genomic signals are rapidly activated within seconds to minutes and involve the activation of several signal transduction pathways, such as MAPKs or PI3K/Akt in E2-sensitive cells [37], [38]. E2-dependent activation of Erk1/2 MAPKs appears to be conserved among a variety of cell lines [39]. In addition, it is emerging that different growth factor receptors, including EGFR, can behave as cell-specific docking sites for E2-activated ER alpha, setting up the basis for the existence of a cross-talk between ER and growth factor receptor signaling [40], [41], [42], [43]. It has been proposed that the mechanisms underlining the estrogen and growth factor receptor cross-talk can be the result of the rapid translocation of ER to the cell membrane, membrane ruffles, and pseudopodia [42], [44], [45]. Membrane-associated and cytoplasmic proteins have also been shown to be important in facilitating ER localization to the plasma membrane and to integrate ER extra-nuclear signaling with other membrane/cytoplasmic signaling pathways. Moreover, the presence of extra-nuclear ER has been suggested to be required in order to mediate E2-induced Erk1/2 MAPK and activation and anti-apoptotic effects in osteoblasts [46]. In addition, it has been reported that extra-nuclear ERalpha upon E2 stimulation can trigger the activation of PI3K by interacting with its regulatory subunit, leading to downstream kinase Akt activation and promoting survival and proliferative pathways [47] Therefore, we can envisage a scenario in which p130Cas-dependent increased survival and MAPK activation observed upon E2 and EGF stimulation might be due at least in part to p130Cas-dependent induction of ER non genomic signaling that synergize on EGFR downstream signaling to MAPKs and Akt.

Finally, it is worth noting that early premalignant breast cancer lesions, such as hyperplastic lesions with atypia and carcinoma in situ, are characterized by a complete or partially filled lumen and that increased signaling through the Erk1/2 MAPKs pathway has been implicated in several types of human breast cancer and in many experimental models of cancer progression [25]. Very little is known about the molecular mechanisms that disrupt normal ductal organization of epithelial cells and understanding these molecular mechanisms may provide new insights on the events that regulate initiation of cancer, and lead to the identification of both molecular markers and drug targets for premalignant disease. Our data clearly indicate that p130Cas up-regulation is implicated in both luminal filling and in tempering Erk1/2 MAPKs levels of activation; two events considered hallmarks of cancer initiation, suggesting that p130Cas over-expression might serve as a bona fide prognostic molecular marker for early breast cancer lesions.

## Supporting Information

Figure S1 **p130Cas over-expression by itself does not affect branching morphogenesis and its expression in wt organoids is not altered upon different stimuli.** (A) Brightfield images of representative wt and p130Tg untreated organoids after 1, 4 and 5 days in the culture medium. (B) p130Cas protein expression does not change upon stimulation with EGF, E2 or EGF+E2.
(TIF)

Figure S2 **E2 treatment of p130Tg organoids alters myoepithelial-luminal architecture and lumen clearance.** Additional representative immunofluorescence images of K14 (red; myoepithelium) and K18 (green; luminal epithelium) staining of wt and p130Tg organoids stimulated with EGF and EGF+E2 at day 5 of culture. Images were taken at 20× magnification.
(TIF)

Figure S3 **p130Cas phoshorylation and expression are not altered following ICI 182,780 and AG4178.** Western blot analysis of phospho-p130Cas (Tyr410) in untreated wt organoids and in organoids pre-treated for 1 hour with ICI 182,780 or AG1478 followed by 5 minutes of E2, EGF or EGF+E2 stimulation. p130Cas and vinculin blots are provided as loading controls. Blots are representative of two independent experiments.
(TIF)

Figure S4 **Effect of the inhibition of Erk1/2 MAPK, EGFR and ER activity in mammary branching morphogenesis.** (A) Brightfield images of representative 5 day wt and p130Tg cultured organoids treated with PD98059, AG1478 and ICI 182,780 and stimulated with EGF or E2/EGF every other day. Images are representative of three independent experiments.
(TIF)
