# Supplementary figures and images for: p130Cas Over-Expression Impairs Mammary Branching Morphogenesis in Response to Estrogen and EGF
Source: PLoS One. 2012 Dec 11;7(12):e49817. doi: 10.1371/journal.pone.0049817 (PMC3519769; doi:10.1371/journal.pone.0049817)

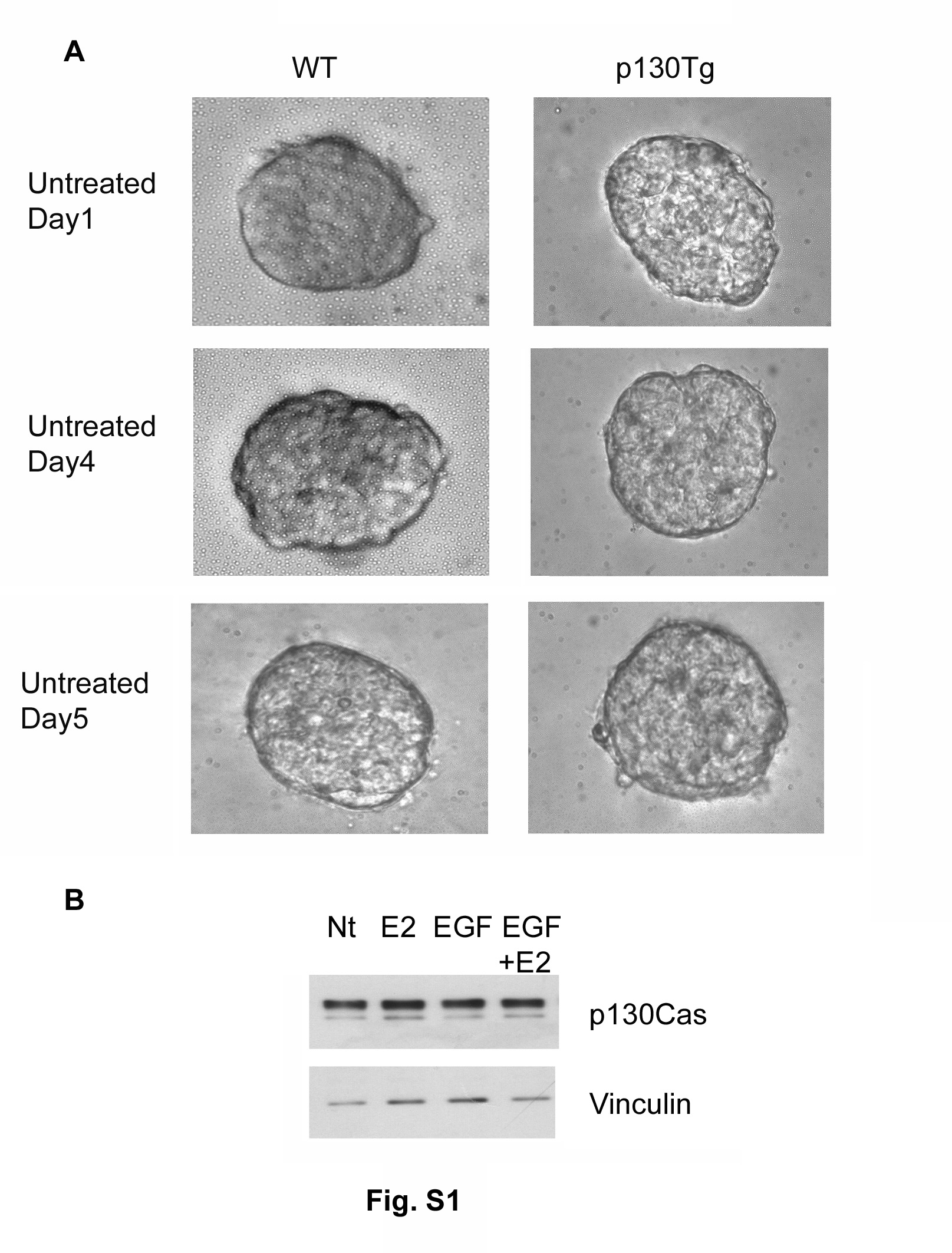

Supplement: Figure S1 — p130Cas over-expression by itself does not affect branching morphogenesis and its expression in wt organoids is not altered upon different stimuli. (A) Brightfield images of representative wt and p130Tg untreated organoids after 1, 4 and 5 days in the culture medium. (B) p130Cas protein expression does not change upon stimulation with EGF, E2 or EGF+E2. (TIF) [file pone.0049817.s001.tif]

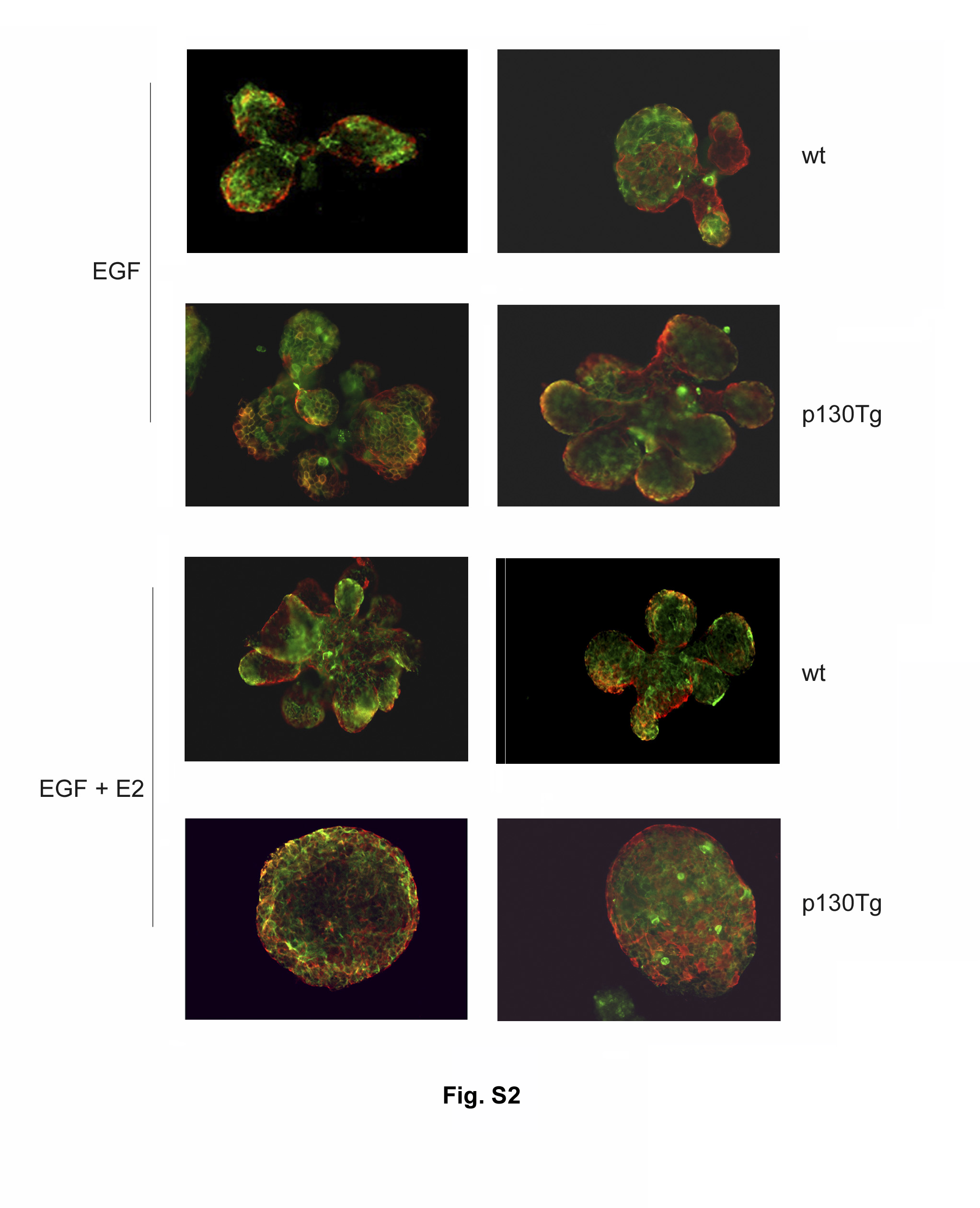

Supplement: Figure S2 — E2 treatment of p130Tg organoids alters myoepithelial-luminal architecture and lumen clearance. Additional representative immunofluorescence images of K14 (red; myoepithelium) and K18 (green; luminal epithelium) staining of wt and p130Tg organoids stimulated with EGF and EGF+E2 at day 5 of culture. Images were taken at 20× magnification. (TIF) [file pone.0049817.s002.tif]

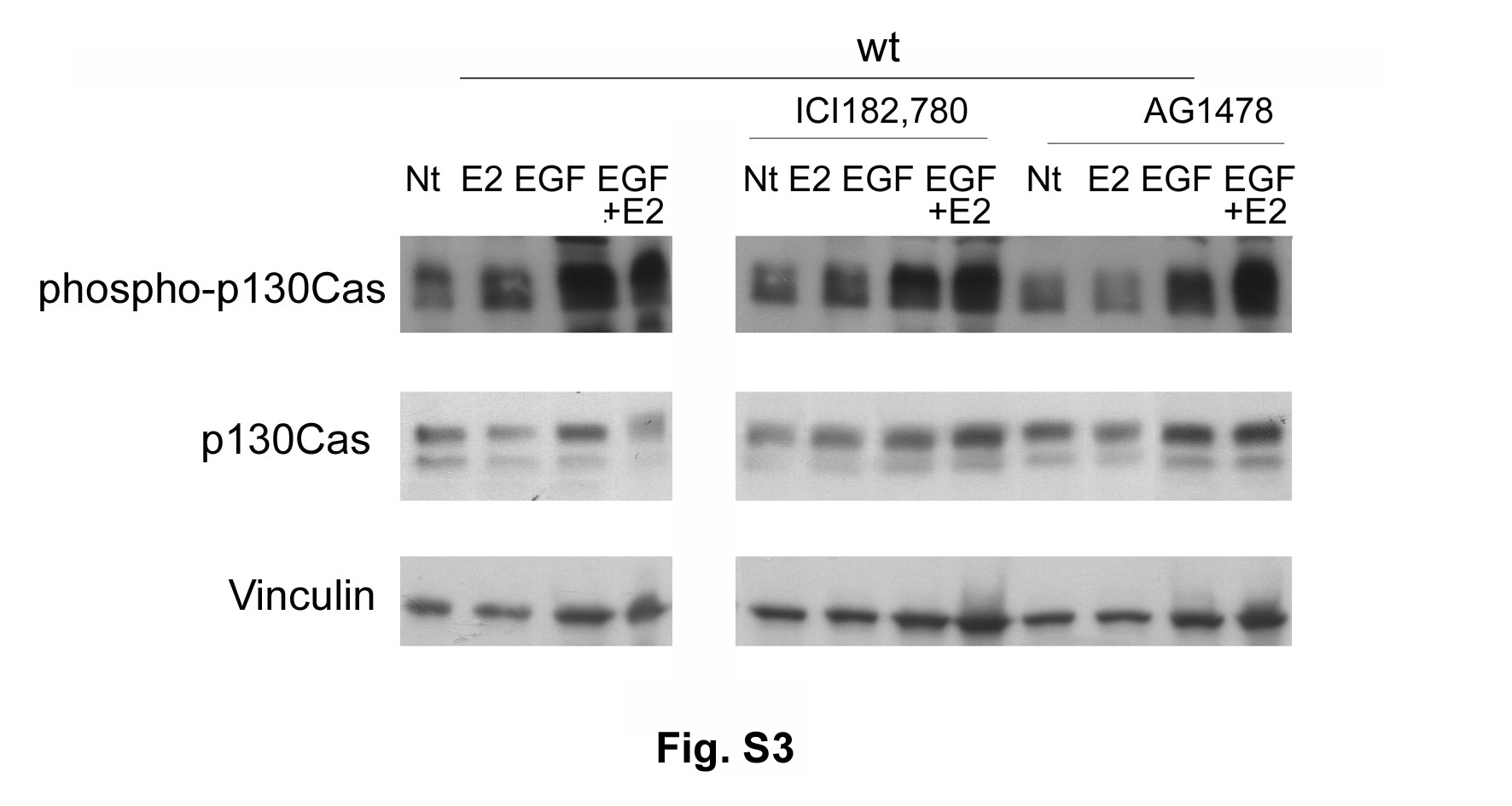

Supplement: Figure S3 — p130Cas phoshorylation and expression are not altered following ICI 182,780 and AG4178. Western blot analysis of phospho-p130Cas (Tyr410) in untreated wt organoids and in organoids pre-treated for 1 hour with ICI 182,780 or AG1478 followed by 5 minutes of E2, EGF or EGF+E2 stimulation. p130Cas and vinculin blots are provided as loading controls. Blots are representative of two independent experiments. (TIF) [file pone.0049817.s003.tif]

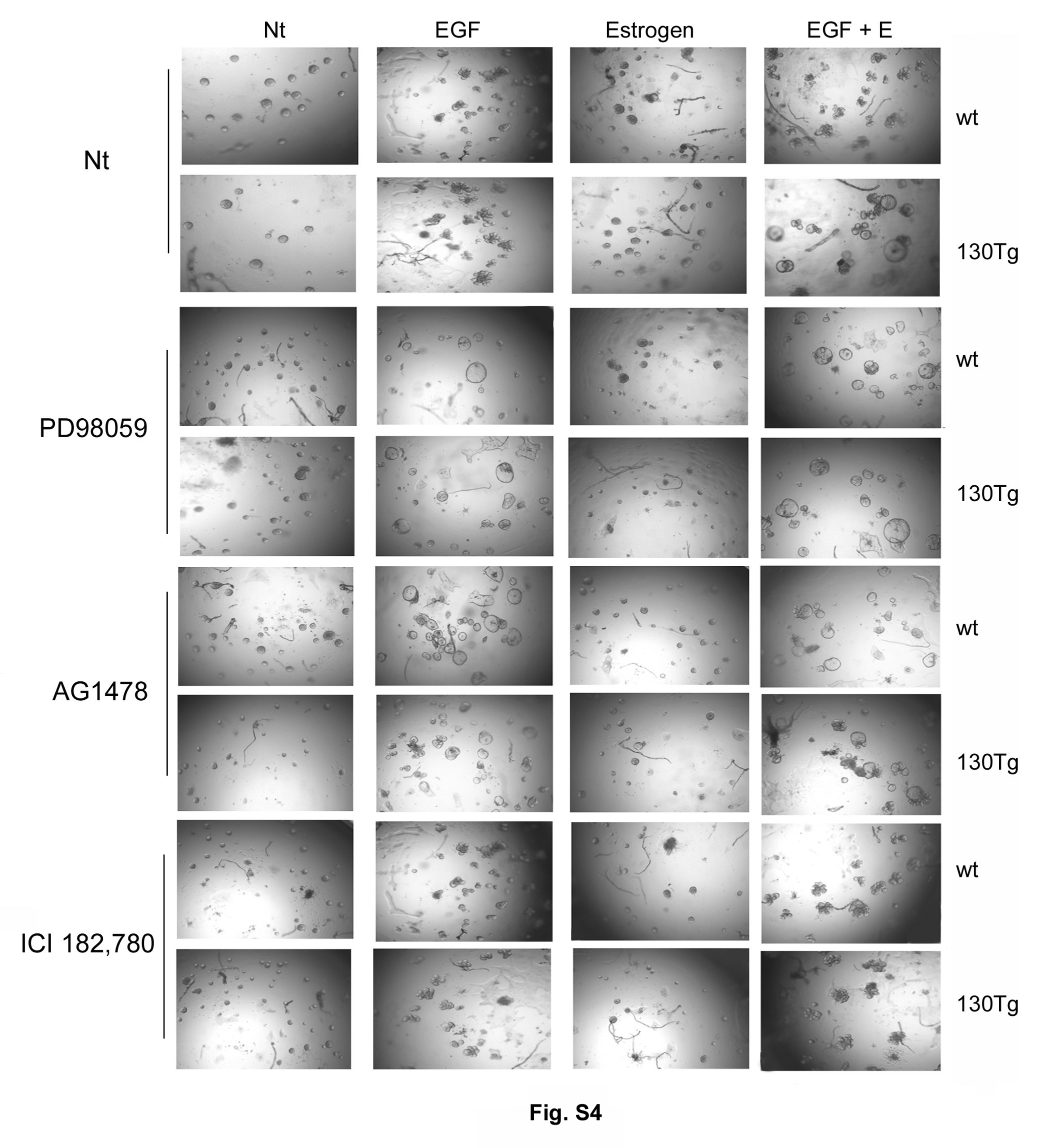

Supplement: Figure S4 — Effect of the inhibition of Erk1/2 MAPK, EGFR and ER activity in mammary branching morphogenesis. (A) Brightfield images of representative 5 day wt and p130Tg cultured organoids treated with PD98059, AG1478 and ICI 182,780 and stimulated with EGF or E2/EGF every other day. Images are representative of three independent experiments. (TIF) [file pone.0049817.s004.tif]
